# Solitary Extraskeletal Giant Osteochondroma of the Ankle in a Four-Year-Old Boy

**DOI:** 10.7759/cureus.39442

**Published:** 2023-05-24

**Authors:** Nickolaos Laliotis, Panagiotis Konstantinidis, Chrysanthos Chrysanthou, Thomas Zarampoukas

**Affiliations:** 1 Orthopaedics, Interbalkan Medical Center, Thessaloniki, GRC; 2 Anatomy and Surgical Anatomy, Aristotle University of Thessaloniki, Thessaloniki, GRC; 3 Pathology and Laboratory Medicine, Interbalkan Medical Center, Thessaloniki, GRC

**Keywords:** benign tumor child, calcified tumor ankle, benign calcified tumor, giant extraskeletal osteochondroma, solitary osteochondroma, trevor disease, dysplasia epiphysealis hemimelica, synovial hyperplasia, extraskeletal osteochondroma

## Abstract

Solitary extraskeletal osteochondromas are rare benign lesions usually located close to a joint and are characterized by the absence of continuity with the adjacent bone. They are usually found in the hand and feet and are extremely rarely reported in the growing skeleton.

In this paper, we describe a four-year-old boy who presented with a solitary calcified tumor in the posterior part of his ankle. We performed a detailed evaluation using plain X-rays, a CT scan, and an MRI, which revealed a well-demarcated calcified tumor that had the characteristics of an osteochondroma but without any continuity with the bones of the ankle joint. The lesion was treated surgically with the excision of a giant osteochondral lesion. Pathological examination revealed mature cartilage at the periphery with cancellous bone in the central part.

Thus, we present the clinical and laboratory investigation of a solitary extraskeletal osteochondroma in the ankle of a four-year-old boy, which is an extremely rare case.

## Introduction

Osteochondromas are common benign lesions that affect the growing skeleton. They are primarily located in the metaphyseal area of long bones. Finding isolated osteochondromas in the bones of the hand or the foot is extremely rare. These osteochondromas have a clear origin in the bone of the foot or the hand, wherein the osteochondroma communicates with the medullary area of the host bone [1-3].

Extraskeletal osteochondromas are rare and arise from the synovium. They are adjacent to a joint and have no continuity with the bone marrow of the proximal bones. They usually present as small multiple osteochondromas in a joint. Although no clear pathogenesis for osteochondromas exists, it is commonly suggested that they arise from synovial hyperplasia [4-7]. The vast majority of extraskeletal osteochondromas affect the hand and feet (82%) and are most commonly presented in patients aged 30-60 years [8]. A distinct form of this disorder is epiphyseal enlargement, which is characterized by the asymmetric growth of the epiphyseal cartilage. This is described as dysplasia epiphysealis hemimelica, commonly known as Trevor's disease. Similar to osteochondromas, these involve projections in the joints that arise from the epiphysis [9,10].

This paper describes a case of a four-year-old boy who presented with a solid lump that was a calcified tumor in the posterior aspect of his ankle. Radiographic evaluation using plain X-rays, a CT scan, and an MRI confirmed the diagnosis of osteochondroma. However, no clear communication was observed with the adjacent bones. The lesion was surgically excised, revealing a loose giant osteochondroma next to the ankle joint with normal synovial tissue. Pathology specimen confirmed the diagnosis of extraskeletal solitary giant osteochondroma. In this paper, we will present the clinical and radiological examination and the pathologic diagnosis for solitary extraskeletal osteochondroma in the ankle of a four-year-old child that, to the best of our knowledge, has not been reported previously. The study was approved by the Ethical Committee of Interbalkan Medical Center (193/14-3-2023).

## Case presentation

The family of a four-year-old boy brought the child to our pediatric orthopedic department for evaluation. His mother had noticed a solid mass in the posterior aspect of the left ankle. The boy was in excellent general health and was participating in all his age-related activities. He was able to walk without any noticeable limp and was performing hip-hop activities that showed no difference between the movements of his right and left legs.

On conducting palpation, we found a solid, round, well-demarked mass without any skin alterations in the posterior medial part of the boy’s right ankle joint. No other bone projections were found in his skeleton. Dorsiflexion and plantarflexion of the affected ankle were restricted by 20 degrees when compared with the unaffected ankle. However, the patient did not feel any pain when pressure was applied to the mass.

**Figure 1:**
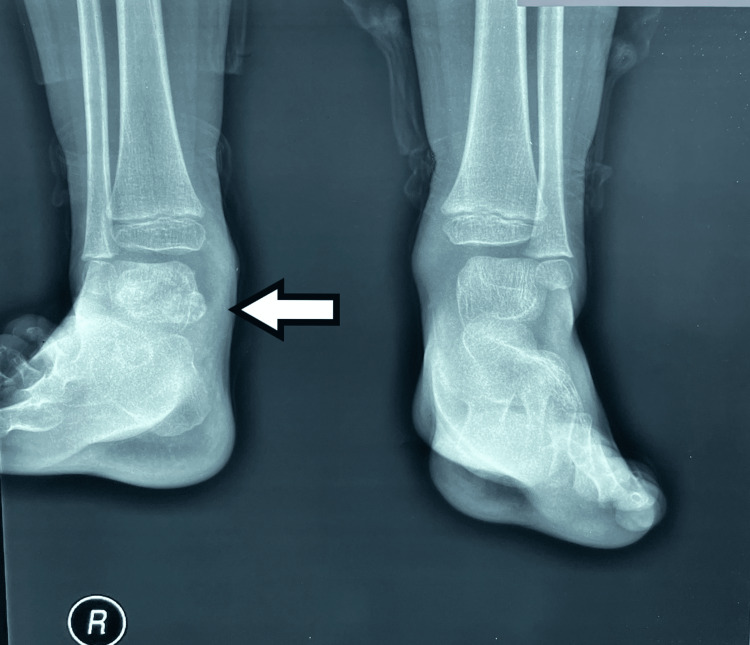
Plain radiographs of the ankle joints in anteroposterior projection. A large calcified lesion, with lobulated margins, is observed adjacent to the posterior margin of the right talus.

**Figure 2:**
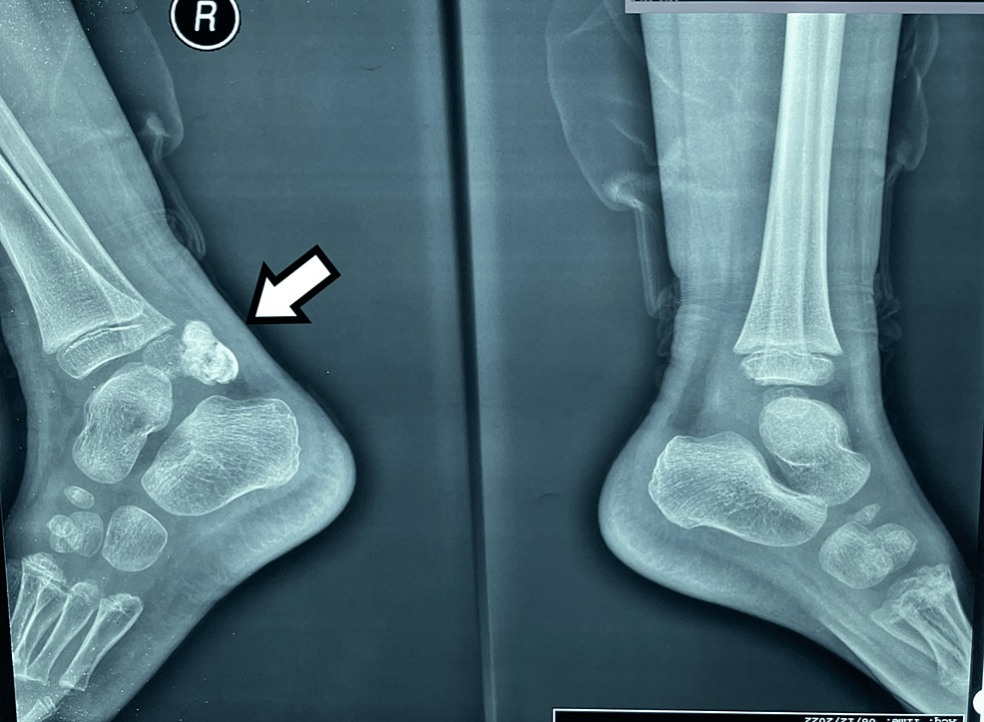
Plain radiographs of the ankle joints in lateral projection. A large calcified lesion, with lobulated margins, is observed adjacent to the posterior margin of the right talus.

An X-ray of the ankle showed a calcified mass in the posterior and medial parts of the ankle joint. The distal tibial epiphysis was of normal shape, and the talus and calcaneum were of normal contour. Calcification was equally distributed in the mass (Figures 1, 2).

**Figure 3:**
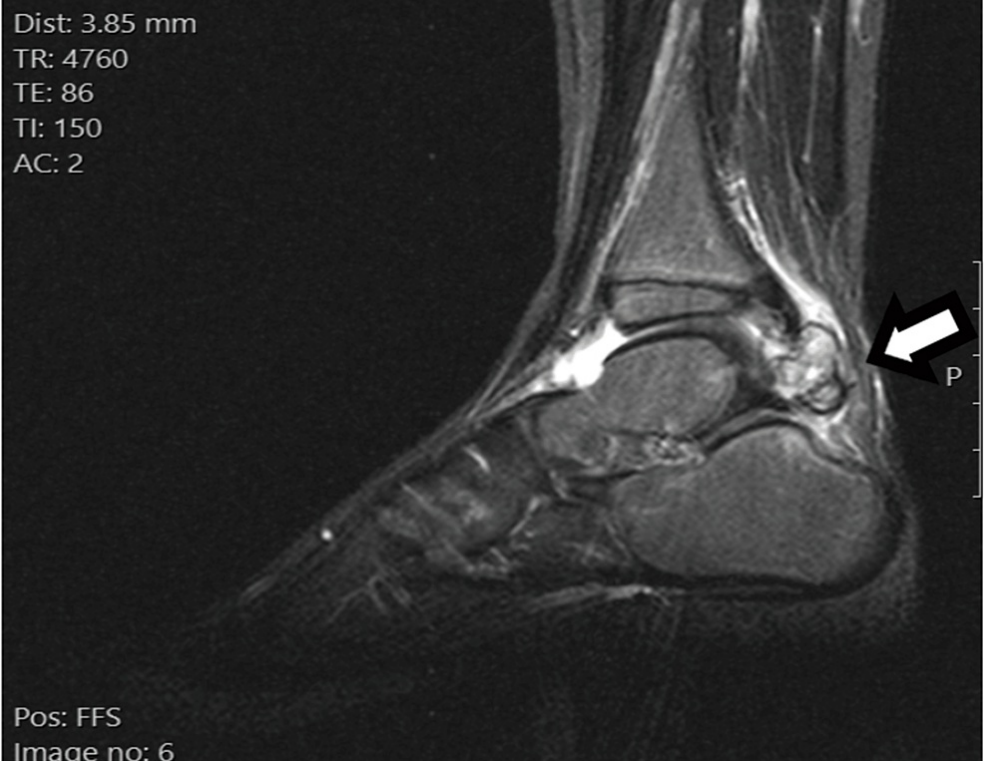
MRI scan of the right ankle sagittal STIR sequence. Short tau inversion recovery (STIR) sequence in the sagittal plane demonstrates a large lobulated mass in the posterior aspect of the ankle joint, which projects in the Kager’s fat pad. The mass has an inhomogeneous appearance, with interchanging areas of high and low magnetic resonance signals. The lesion is in close proximity to the cortex of the posterior surface of the talus, without clear evidence of a bony protuberance that connects the lesion with the host bone. An increased amount of fluid is observed in the ankle joint.

**Figure 4:**
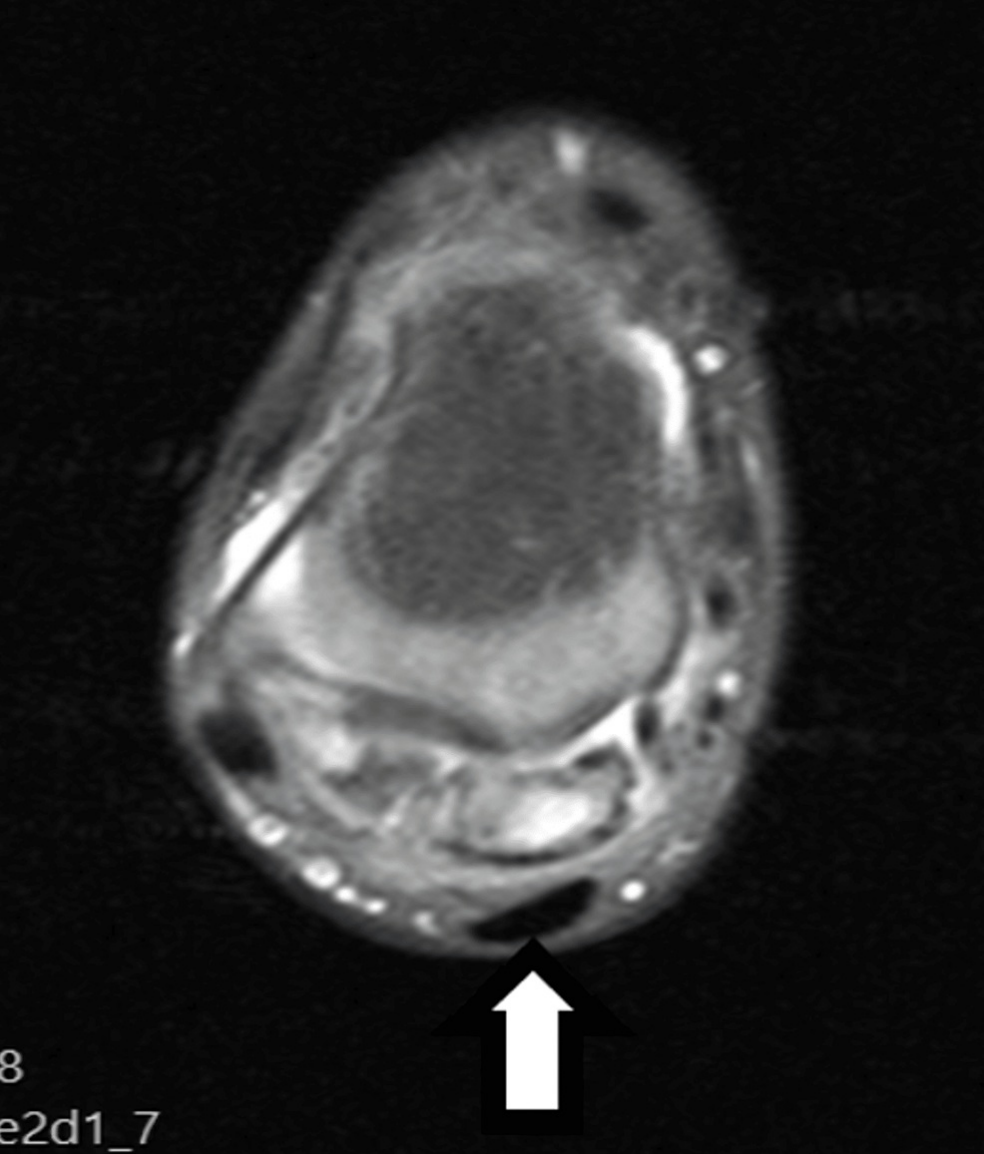
MRI scan of the right ankle, axial proton density (PD) fat saturated. Proton density (PD) sequence with fat suppression in the axial plane also demonstrates the relationship of the mass with the adjacent anatomic structures.

**Figure 5:**
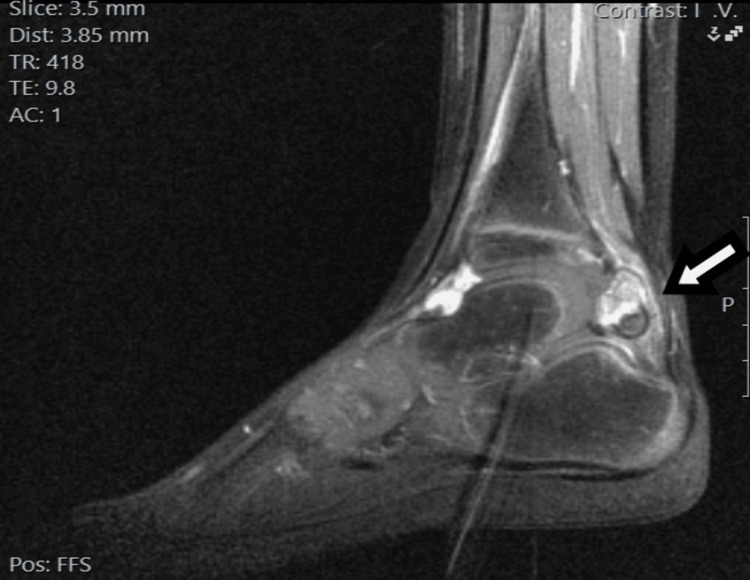
MRI scan of the right ankle T1C sequence. T1C sequence in the sagittal plane after the intravenous administration of paramagnetic agent shows inhomogeneous enhancement of the lesion.

An MRI scan of the ankle revealed a heterogeneous mass adjacent to the posterior margin of the talus, with areas of intermediate- and high-signal intensity on short tau inversion recovery (STIR) sequences. Inhomogeneous enhancement was observed after intravenous administration of a paramagnetic agent. We could not observe a clear continuity either with the posterior aspect of the tibial or fibular epiphysis or with the posterior aspect of the talus. The synovium was normal without any signs of thickened bursa adjacent to the calcified tumor (Figures 3-5).

**Figure 6:**
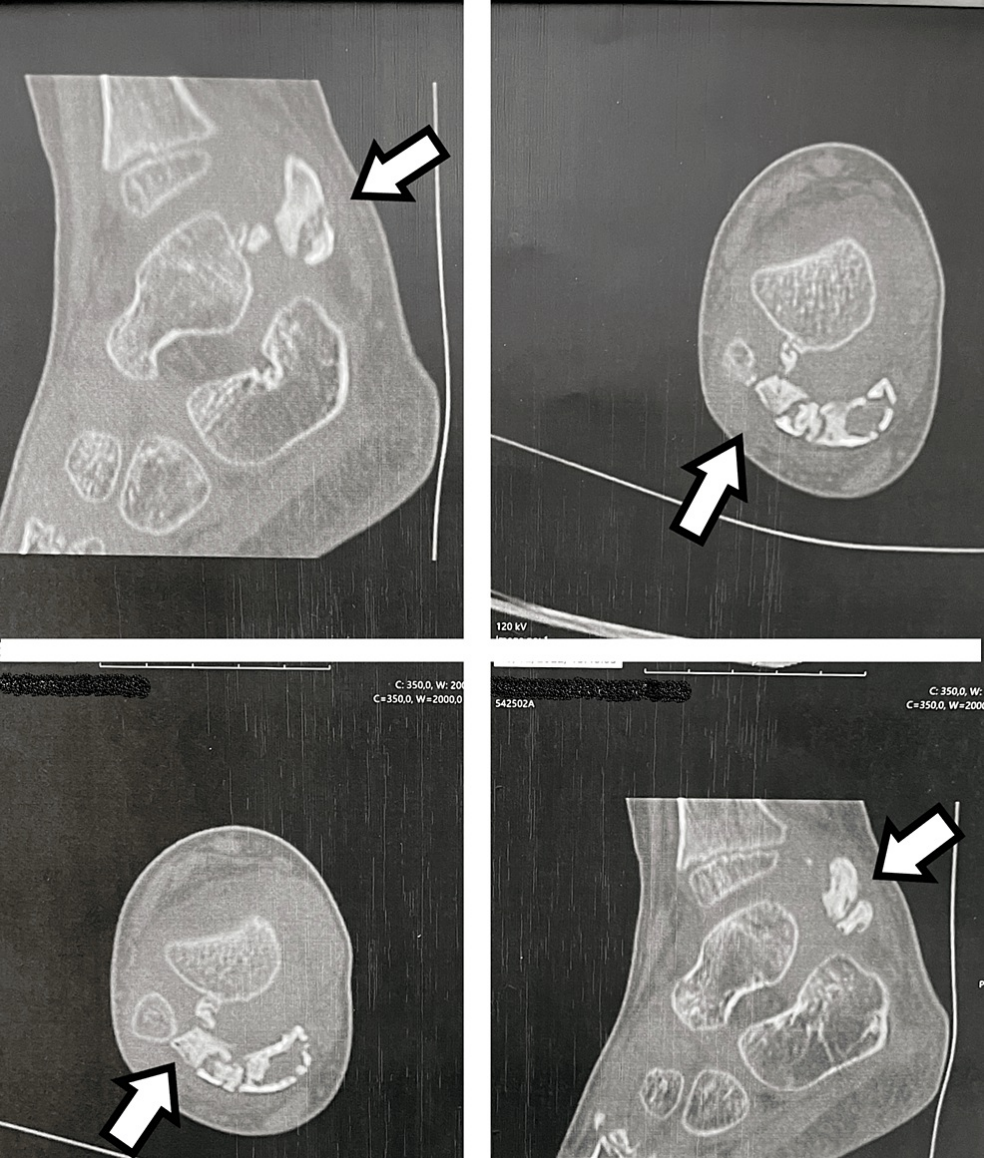
CT scan of the right talus with multiplanar reconstruction. There is extensive calcification of the mass. A small calcified stalk is observed between the lesion and the talus, but no clear continuity of the stalk with the talus is confirmed.

To further evaluate the relationship of the lesion with the adjacent bones, we performed a CT scan. The scan revealed that the lesion was largely calcified. A single lesion was found with dimensions of 19.6 mm longitudinal and 13.7 mm transverse. A small projection was noted in the posterior aspect of the talus; however, it had no clear continuity with the calcified mass. Based on these findings, we diagnosed the type of osteochondroma to determine whether it was extraskeletal osteochondroma or Trevor's disease. The differential diagnosis included myositis ossificans or calcinosis. Atypical extraskeletal Ewing sarcoma or chondroblastoma were exceptions for the final diagnosis (Figure 6).

**Figure 7:**
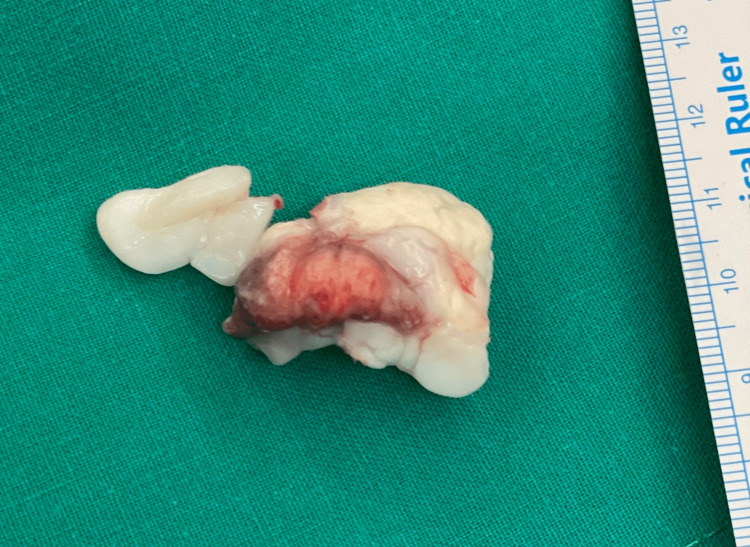
A gross image of the osteochondroma with cartilaginous cap and centrally located bone.

**Figure 8:**
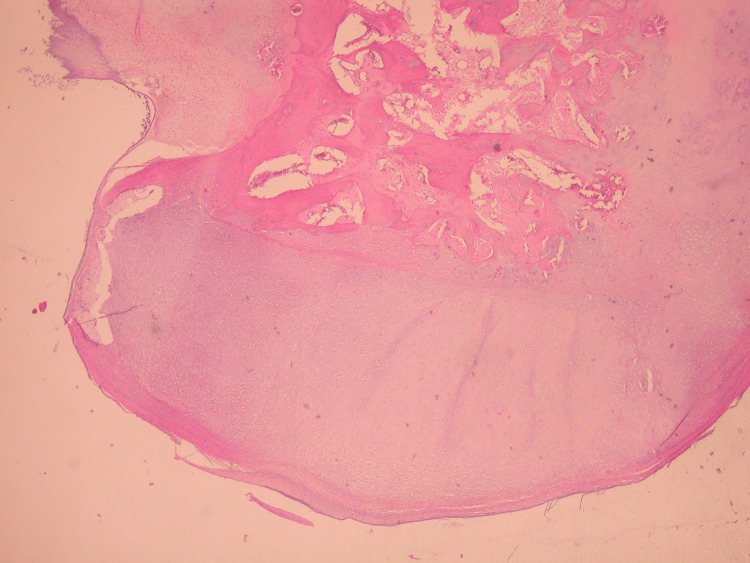
Typical morphology of osteochondroma. Zone of mature cartilage at the periphery and centrally cancellous bone (hematoxylin & eosin, x25).

**Figure 9:**
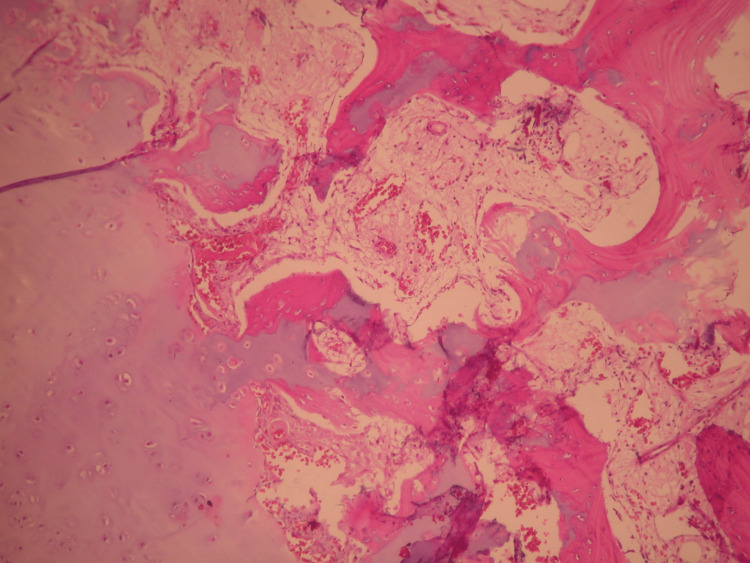
Mature cartilage with chondrogenic ossification and progression to the cancellous bone with fatty marrow (hematoxylin & eosin, x25).

The boy was surgically treated, employing a posterior medial approach to the ankle. Upon opening the synovium of the ankle joint, we found a typical osteochondroma appearing as a poly-lobulated dense mass that was covered starting from the hyaline cartilage and became free in the ankle joint. It could be easily retrieved from the joint as it was not connected to any of the adjacent bones. The posterior aspect of the talus appeared normal. The size of the cartilaginous mass matched the dimensions revealed in the CT scan. The joint had no other mass, and the synovium appeared normal. We sent the mass and a small piece of the synovium to the pathology department. The pathology specimen confirmed the diagnosis of solitary extraskeletal osteochondroma. At the periphery, a zone of mature cartilage was discovered, while the cancellous bone with fatty marrow was found centrally (Figures 7-9).

The boy had an uneventful recovery. We reviewed him a week, a month, and three months post-surgery. We also performed a plain X-ray, which revealed the normal configuration of the ankle joint. The boy has returned to all his activities.

## Discussion

Reports for extraskeletal solitary giant osteochondromas are rare in the extant literature. Synovial giant osteochondromas are primarily reported to affect adults. They originate from the synovium and are presented as multiple loose bodies in the joint [7,11-13].

Osteochondromas are commonly found incidentally, resembling a smooth solid mass. They do not cause pain but occasionally present a snapping symptom upon increasing in size. In our patient, the extra-articular position of the mass was not affecting the ankle movements; therefore, the child exhibited no movement disorders, such as limping. Osteochondroma is the most common benign lesion of the metaphysis of long bones. Solitary osteochondromas are extremely rare in hand and foot bones. The hallmark for the diagnosis of osteochondroma is the presence of a bony protuberance in direct continuity with the medullary cavity of the adjacent bone of origin, with either a broad base (sessile osteochondroma) or a thin base (pedunculated osteochondroma). The bone marrow of the lesion should follow the magnetic resonance (MR) signal of the parent bone’s bone marrow in all sequences. The cartilaginous cap is well identified in MRI scans with a high MR signal on T2-weighted sequences due to high water content [14-16].

Solitary synovial osteochondromas have a distinct radiological appearance. An X-ray evaluation reveals the presence of multiple periarticular calcified parts that present as loose bodies without bone erosion [5]. These are found in all parts of the joint. Solitary synovial osteochondroma has been described to affect the knee, buttocks, and ligamentum teres. A few studies have focused on solitary extraskeletal osteochondromas in the region of the ankle joint affecting young patients (nine to 18 years of age) [4-6,8,17,18].

Evidence suggests that synovial osteochondromas evolve in three phases. The first phase is characterized by active synovial disease; the second phase involves the formation of loose bodies; and in the final phase, only osteochondral bodies without synovial disease can be found [1,19]. Milgram and Dunn used the term para articular extraskeletal osteochondroma, distinct from synovial chondromatosis, to describe these lesions as consisting of mature bone tissues that are covered cartilage onward and are without connection with the adjacent bone. Reith et al. further described them as a single dominant mass similar to conventional osteochondroma, which arises from nonsynovial tissue. The peak incidence of this disorder is reported in the fourth to seventh decade of an individual's life. The knee joint is the most frequently affected, while the ankle is affected in 5% of the patients [19-21].

Solitary osteochondromas adjacent to a joint must be distinguished from the asymmetric overgrowth of the epiphysis, known as the Trevor disease. This usually affects the distal tibial epiphysis. Continuity with the epiphysis is observed, and it usually presents as spurs with endochondral ossification. It is covered cartilage onward, and projections are present in the ankle joint. Trevor disease can be localized, which affects a single epiphysis with more than one epiphysis in the same limb, or generalized, affecting the entire limp [9,10,20-23].

In our patient, we could not detect any communication between the calcified mass and the adjacent bones. Other diseases such as myositis ossificans circumscripta, tophus calcification, and extraskeletal osteosarcoma were unlikely as the radiological evaluation had the characteristics of osteochondroma; however, they were included in the differential diagnosis. Solitary extraskeletal osteochondromas are histologically similar to osteochondromas that have communication with the affected bone [14,15].

## Conclusions

Surgical treatment is the treatment of choice for the removal of osteochondromas and appropriate pathological examination. We performed an open procedure since the mass was protruding in the posterior aspect of the ankle and was not intra-articular-located. Our patient presented a loose giant osteochondroma without any connections with the adjacent bones.

As our patient is young, we are regularly following him up at six-month intervals to evaluate the possibility of a relapse.
